# MicroRNA-Regulated Proinflammatory Cytokines in Sarcopenia

**DOI:** 10.1155/2016/1438686

**Published:** 2016-06-13

**Authors:** Jingjing Fan, Xianjuan Kou, Yi Yang, Ning Chen

**Affiliations:** Hubei Key Laboratory of Exercise Training and Monitoring, Hubei Provincial Collaborative Innovation Center for Exercise and Health Promotion, College of Health Science, Wuhan Sports University, Wuhan 430079, China

## Abstract

Sarcopenia has been defined as the aging-related disease with the declined mass, strength, and function of skeletal muscle, which is the major cause of frailty and falls in elders. The activation of inflammatory signal pathways due to diseases and aging is suggested to reveal the critical impact on sarcopenia. Several proinflammatory cytokines, especially interleukin-6 (IL-6) and tumor necrosis factor-alpha (TNF-*α*), play crucial roles in modulation of inflammatory signaling pathway during the aging-related loss of skeletal muscle. MicroRNAs (miRNAs) have emerged as the important regulators for the mass and functional maintenance of skeletal muscle through regulating gene expression of proinflammatory cytokines. In this paper, we have systematically discussed regulatory mechanisms of miRNAs for the expression and secretion of inflammatory cytokines during sarcopenia, which will provide some novel targets and therapeutic strategies for controlling aging-related atrophy of skeletal muscle and corresponding chronic inflammatory diseases.

## 1. Inflammation System during Sarcopenia

### 1.1. Sarcopenia-Related Changes in Immune System

Sarcopenia is present in approximately 5–13% of persons over the age of 60 years and defined as the loss of skeletal muscle mass and strength with progressive decline in mobility and function [1]. The pathogenesis of sarcopenia is associated with many intrinsic and extrinsic factors, including proinflammatory cytokine accumulation, oxidative stress, mitochondrial dysfunction, insulin resistance, and aging-related loss of anabolic hormones and motor neuron end plates [2]. The loss of skeletal muscle results from an imbalance of protein metabolism, which is the dynamic balance between protein degradation and protein synthesis [3]. The protein degradation systems in skeletal muscle are modulated by a coordinated network of signaling pathways activated [4] or suppressed by hormones and cytokines; therefore, catabolism is stimulated by a variety of proinflammatory cytokines, glucocorticoids, and reactive oxygen species (ROS) [5, 6].

**Figure 1:**
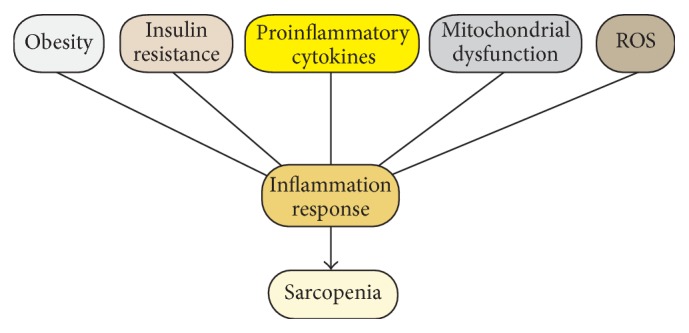
The interplay between sarcopenia and chronic inflammation. The boxes represent domains known to influence the maintenance of sarcopenia and inflammation response in aging organisms. Abbreviations: ROS, reactive oxygen species.

There is no simple mechanism to explain aging-associated loss of skeletal muscle. It is important to note that impaired cellular immune function combined with low-grade inflammation represents a continuous impact in aging process [7]. Although aging is associated with prolonged inflammatory activity that is mainly attributed to progressively worsening muscle weakness, it is unclear whether these processes are cross-talked. Molecular signals and pathways connecting inflammatory system and muscle degeneration may be the key to reveal the interactions responsible for the progression of sarcopenia. The interplay seems to exist between the mass loss of skeletal muscle and elevated systemic inflammation (Figure 1). Sarcopenia is a complex process with a subclinical state of inflammation driven by proinflammatory cytokines and oxidative stress, which increases the infiltration of immune cells into injured muscles. In turn, inflammation aggravates muscle loss and fat accumulation in the aging skeletal muscle and further decreases muscle function and physical activity [8]. The increase in chronic inflammation response associated with high-level proinflammatory mediators as the extension of age has been considered as one of the diagnostic hallmarks and a significant contributor to aging-related atrophy of skeletal muscle [9]. The transcription factor, nuclear factor-*κ*B (NF-*κ*B), has been considered as an important mediator underlying the relationship between inflammation and aging [10, 11]. The correlation between inflammation and sarcopenia exists as a possible linkage describing the effect of inflammation on the balance between protein anabolism and catabolism, with the presence of CD68+ macrophage infiltration [12].

It is worth noting that substantial evidence also demonstrates the connection between obesity and sarcopenia, namely, “sarcopenic obesity” [13]. In fact, obesity always plays an important role in sarcopenia, by the way of adding inflammatory burden [14]. During obesity, adipose tissue is characterized by a chronic inflammatory state, through the release of numerous proinflammatory cytokines including tumor necrosis factor-alpha (TNF-*α*), interleukin-6 (IL-6), and interleukin-1 beta (IL-1*β*) as the factors largely responsible for insulin resistance in obese adipose tissue combined with aging-related skeletal muscle loss [15].

Moreover, impaired mitochondria can be both the reason and the consequence of inflammation during aging [16]. Increasing evidence shows that mitochondria may contribute to inflammation via ROS production, NF-*κ*B activation, calcium homeostasis, impaired autophagy, and ATP deficiency [17, 18]. Dysfunctional mitochondria are able to modulate aging-related inflammatory processes through direct activation of NLRP3 inflammasome, which can correspondingly result in the activation of caspase-1 or redox-sensitive inflammatory signaling pathways, thus leading to the production of IL-1*β* and IL-18 [19, 20]. It should be noted that increasing ROS could stimulate the activation of NF-*κ*B via NF-*κ*B-inducing kinase (NIK) and I*κ*B kinase *α* and *β* (IKK*α*/*β*) [21, 22]. Since the increased redox activation in the presence of transcription factor NF-*κ*B, excessive ROS generation plays an important role in impaired mitochondrial function and oxidative capacity and accelerates the aging process of skeletal muscle [23].

### 1.2. Proinflammatory Cytokines Associated with Sarcopenia

As described above, sarcopenia is a common feature in the elderly and mainly related to the release of inflammatory mediators from damaged tissue. These responses are controlled by a combination of various cytokines responsible for inflammatory pathways [10, 24]. Given that the inflammatory response is a complex system, cytokines are important not only as the indicators for mediating chronic inflammatory state through increasing protein degradation and reducing protein synthesis, but also as the mediators for controlling muscle wasting by directly targeting muscle tissue [25]. In particular, proinflammatory cytokines are well known to impinge on protein metabolism in skeletal muscle and result in the activation of catabolism signals or upregulate inflammatory pathways such as NF-*κ*B and STAT3, thus finally leading to the increased activation of ubiquitin-proteasome and autophagy system [26, 27]. The chronic inflammatory aging process depends not only on increased expression of proinflammatory factors, but also on reduced levels of anti-inflammatory factors such as IL-10, one of the anti-inflammatory cytokines [28]. Since the presence and function of cytokines have been demonstrated in the pathogenesis of sarcopenia, their origins and types must be identified. In fact, cytokines can be secreted by various types of cells like inflammatory and stromal cells, as well as skeletal muscle cells. In skeletal muscle, mature myofibers make up cellular mass, and myotubes express mRNAs of various cytokines. The constitutive expression of cytokines is generally stronger in differentiated myotubes compared with myoblasts, which usually results in inconspicuous change in cytokine release to stimuli [29, 30]. Accumulating evidences over the past decade have demonstrated that those proinflammatory cytokines such as TNF-*α*, IL-6, and C-reactive protein (CRP) cause a significant increase in aging skeletal muscle cells and play a key role in the complex network of inflammatory signals in charge of muscle homeostasis connected with aging-related disability and mortality [31, 32].

#### 1.2.1. IL-6 and CRP

IL-6 which is coined “cytokine for gerontologists” [33] has originally been classified as a prototypical proinflammatory cytokine to exhibit marked pleiotropy, and its anti-inflammatory property has also been identified later [34, 35]. IL-6 plays an important role in the pathogenesis of several chronic diseases including sarcopenia by regulating inflammatory and metabolic functions [36]. IL-6 signaling involves the binding to the membrane-bound IL-6 receptor in skeletal muscle and the activation of downstream signaling pathways including STAT3, MAPK/ERK, p38, myostatin, and FoxO3 pathways [37–40]. Additionally, IL-6 has confirmed to have the function of activating AMPK and/or phosphatidylinositol-3-kinase (PI3K) and regulating the metabolism in skeletal muscle [41, 42]. The overexpression of IL-6 can result in reduced body mass and impaired insulin-stimulated glucose uptake in mouse skeletal muscle [43]. Furthermore, the infusion of IL-6 in skeletal muscle can reduce the phosphorylation of S6K1, which is activated by Akt/mTOR and associated with the inhibition of anabolic process [44]. A comparative analysis of cytokine levels has confirmed the upregulation of proinflammatory IL-6 and CRP in the elderly, along with increased risk for the loss of skeletal muscle mass and strength [45]. According to a recent study, the serum high-sensitivity CRP (hs-CRP) levels in the obesity only and in the sarcopenic obesity groups are significantly higher than that in the normal group after multivariate adjustments, which provides the evidence that obesity and sarcopenic obesity are associated with increased levels of serum hs-CRP among males [46].

#### 1.2.2. TNF-*α*

TNF-*α* is a cytokine implicated in the metabolic disturbance of chronic inflammation, with the formation of IL-1, which has been identified as a circulatory factor to increase gluconeogenesis, lipolysis, and proteolysis, accompanied with the decrease in protein, lipid, and glycogen synthesis in skeletal muscle [47]. During the stages of muscle regeneration, TNF-*α* and IL-1 are observed in injured muscle with an accumulation of macrophages [48, 49]. Also, TNF-*α* and IL-1 have been confirmed to promote IL-6 secretion through activating NF-*κ*B in cultured skeletal muscle cells [50]. Several previous studies have confirmed that TNF-*α* at the elevated level can increase catabolism in skeletal muscle by suppressing Akt/mTOR pathway [51]. TNF-*α* and its soluble receptors have been described as the important contributors or biomarkers for the loss of mass and strength in aged skeletal muscle [52]. There is a strongly negative correlation between protein breakdown and TNF-*α* concentration in the elderly [53]. Injecting TNF-*α* into mice has revealed the activation of ubiquitin-proteasome system and the decrease of skeletal muscle function [54].* In vivo*, the synthesis rate of myosin heavy chain protein is correlated negatively with the expression of TNF-*α* in skeletal muscle. TNF-*α* induces skeletal muscle loss through increased myofibrillar protein degradation and cell apoptosis, thus resulting in muscle atrophy and the inhibition of muscle regeneration following injury [55, 56]. Additionally, it seems that TNF-*α* may antagonize the anabolic effect of insulin growth factor-1 (IGF-1), due to the development of growth hormone resistance, which decreases both circulating and muscular IGF-1 [57, 58]. Recent studies have shown that G/A-308 TNF-*α* polymorphism as a marker of sarcopenia in normal weight obese syndrome, suggesting the importance of TNF-*α* in the diagnosis of sarcopenia [59]. It is important to note that an increase in TNF-*α* alone is not sufficient to cause muscle atrophy. The upregulation of NF-*κ*B can cause muscle atrophy in rodents and contribute to the progressive muscle loss of advancing age [60].

#### 1.2.3. Other Inflammatory Cytokines

Other mediators such as interferon-*γ* (IFN-*γ*) are produced in the microenvironment of skeletal muscle and play a critical role during myogenesis. Moreover, IL-15 [61] is usually mentioned among paracrine effectors, while cytokines such as irisin [62] and myonectin [63] are able to induce anti-inflammatory cytokines (IL-1 receptor antagonist and IL-10), especially during contraction and aging conditions [64]. The levels of TLR4 protein and IL6, IL10, and IL15 mRNA expression are increased after short-time bed rest in healthy older adults, while the levels of IFN-*γ* and macrophage inflammatory protein-1-beta (MIP-1*β*) are elevated in aging skeletal muscle [65]. Furthermore, peroxisome proliferator-activated receptor gamma coactivator-1-alpha (PGC-1*α*) is regarded to have an anti-inflammatory function by inhibiting the function of FoxO3, which could promote inflammatory cytokine expression and downregulate antioxidant enzyme expression in aging muscle [66]. The reduced expression of PGC-1*α* results in a low level and a systemic inflammatory response to exhibit negative impacts on skeletal muscle [67]. Inversely, the upregulated PGC-1*α* can reduce the activity of NF-*κ*B, which contributes to the inhibition of proinflammatory cytokines to be benefit for the prevention of mass and strength loss of skeletal muscle and functional decline of other organs, as well as the ultimate impact on homeostasis in human body [68, 69].

Proinflammatory cytokines have a wide variety of roles in inflammation systems and may be the important factor predisposing to muscle catabolism response in the elderly. However, the roles of these cytokines in aging skeletal muscle are still not fully understood. Furthermore, the tissue-specific inflammatory signaling pathways in response to cytokines along with the elevation of systemic cytokines are important elements to be considered.

## 2. MicroRNA- (miRNA-) Mediated Inflammation and Skeletal Muscle Loss

### 2.1. Biogenesis and miRNAs

miRNAs are short noncoding RNAs with approximately 22 nucleotides in length and are involved in the complex posttranscriptional regulatory networks and the maintenance of healthy cellular functions such as growth, development, and metabolism [70]. Skeletal muscle is the most abundant tissue in human body, comprising 40–50% of body mass. It is estimated that approximately 60% of human genes are regulated by miRNAs, suggesting that highly enriched miRNAs in skeletal muscle play important roles in biological processes by gene silencing, including aging process [71]. The functions of miRNAs can be achieved either by suppressing the translation of target messenger RNAs (mRNAs) or by promoting the degradation of mRNAs, thereby providing a powerful and sensitive regulator to tune gene expression and cell functions during the aging process of skeletal muscle [72].

miRNAs, from noncoding RNA genes or within protein-coding genes, are transcribed into primicroRNAs by RNA polymerase II or polymerase III in some cases and subsequently embedded into premicroRNA hairpins like RNA duplex by Drosha [73, 74]. PremicroRNA hairpins are exported from nucleus by exportin-5 and processed into double-stranded mature miRNAs by Dicer in combination with its RNA-binding cofactor [75]. After Dicer-mediated maturation, the miRNAs orient the RISC complex with the removal or the preservation of one strand as the guide strand and preferentially load on the RISC complex in position at regulatory sequences in target genes [76].

Although the precise mechanisms for miRNA targeting and activity still remain to be fully explored, miRNA activity appears to be largely dependent on its binding capacity to the target mRNA molecule [77–79]. Generally, mRNAs contain a predicted binding site in the 3′ untranslated region (UTR), less commonly, in the 5′ UTR, and many mRNAs contain multiple potential binding sites. There are 2 known binding types for miRNAs [80]. According to the binding complementarity of the seed sequence, Argonaute proteins such as Ago-2 can directly cleave messenger RNA and normally repress gene expression by targeting the mRNA for degradation (“complete match,” RISC binds to mRNA with perfect match) or by mediating translation inhibition or mRNA deadenylation leading to mRNA destabilization at the condition with mismatches between mRNA sequence and the RISC (“incomplete match,” RISC binds to mRNA with some mismatches) [81].

However, there are still unsettled questions regarding miRNA-binding rules, thereby resulting in a lack of consensus in previous studies. Establishing direct cause-and-effect links between miRNAs and mRNA targets is a key to understand underlying molecular mechanisms behind health and diseases and to develop effective targeted therapeutic strategies. Now that miRNAs have multiple gene targets, each target may be regulated by a suite of miRNAs. The roles of miRNAs in inflammation and sarcopenia have only been initially explored and future investigations will unravel their roles in immunity and metabolism.

### 2.2. miRNA-Regulated Signal Pathways for Proinflammatory Cytokines during Sarcopenia

According to the analysis of miRNA expression profiling, miRNAs are critical regulators for both proinflammatory cytokines and skeletal muscle function [82, 83]. In order to elucidate which miRNA is important in the production of proinflammatory cytokines in aging-related muscle wasting, mRNA targets and specific roles in regeneration and protein synthesis in skeletal muscle need to be established. Several tissue-specific miRNAs are known to be associated with the aging of skeletal muscle, which are named as myomiRNAs and consistently identified, including miR-1, miR-133, miR-206, miR-208, miR-486, miR-431, and miR-499 [84–86]. These myomiRNAs can induce significant effects on development and myogenesis of skeletal muscle by targeting myogenic factors such as SRF, MEF2, and myostatin [87]. Local injection of miR-206 can accelerate muscle regeneration [88], and miR-133 can promote the proliferation of myoblasts, while miR-1 can suppress the proliferation of myoblasts [83]. Although there is no obvious difference in the expression of mature miR-1, miR-133, or miR-206 in skeletal muscle from younger adults [89], an increased expression of these primary miRNAs can be observed during aging, and the effect of anabolic stimulus on the levels of these miRNAs can be perturbed in the elderly. On the other hand, many studies have implicated the regulation of inflammatory response through inflammatory miRNAs such as miR-155 and miR-146a, suggesting their roles in the immune system [90]. Since inflammation is rather a broad concept, there are some overlaps between miRNAs involved in inflammation and aging [91]. Therefore, cytokine-associated miRNAs appear to have central roles in both inflammation and sarcopenia.

A recent RNA sequencing study has demonstrated the differential expression of miRNAs in skeletal muscle from old and young rhesus monkeys [92]. miR-181a, with its role in tuning the threshold of T-cell receptor (TCR) signaling originally described [93], not only acts as a myomiRNA, but also impacts inflammatory system. It can downregulate sirtuin 1 (Sirt1) gene expression as a regulator so that the expression of miR-181a and its target gene is disrupted in aged skeletal muscle [94]. Moreover, based on earlier reports showing proinflammatory cytokines such as TNF-*α*, IL-6, IL-1*β*, and IL-8 as the proposed targets of miR-181a, the reduction of miR-181a can be responsible for an increase in the abovementioned proinflammatory cytokines in skeletal muscle during aging process [95]. Besides, TNF-*α* and IL-1*β* are significantly negatively correlated with decreased expression of myomiRs and can suppress the differentiation of C2C12 myoblasts to myocytes/myotubes through NF-*κ*B, JAK/STAT, MAPK p38, or other key pathways [96].

A newly discovered proinflammatory cytokine, TNF-like weak inducer of apoptosis (TWEAK), belongs to TNF family and has revealed the function for causing muscle atrophy [97]. One of the mechanisms proposed for the induction of skeletal muscle wasting by TWEAK is regulating differential expression of several growth-related miRNAs, including miR-1, miR-23, miR-133a, miR-133b, and miR-206 in C2C12 myotubes; however, it can reduce miR-1, miR-133a, and miR-133b only in mouse skeletal muscle [98]. While the treatment with TWEAK regulates several miRNAs involved in the growth of skeletal muscle, it is not known whether their regulation is a cause of muscle wasting or a compensatory response to prevent further muscle wasting.

Let-7 miRNA, the first known human miRNA, has been reported to be critical for promoting differentiation and inhibiting cellular proliferation [99]. The elevation of let-7 miRNA may be responsible for the damage-repairing capability through the activation and proliferation of satellite cells in skeletal muscle from the elderly, therefore contributing to the attenuated regenerative capacity of skeletal muscle in the elderly [100]. Moreover, let-7 miRNA can inhibit the secretion of inflammatory cytokine IL-13 in human myotubes [101]. Recent study has also found that the overexpression of miRNA let-7c can inhibit LPS-induced production of TNF-*α*, IL-6, and IL-1*β* by inhibiting the phosphorylation of STAT3 [102]. Compared to younger individuals, skeletal muscle from older individuals shows a significant elevation in let-7b and let-7e under resting conditions, suggesting the involvement of these miRNAs in regulating cell cycle based on let-7 target genes [100].

miR-146a, which negatively regulates the expression of IL-1*β* and IL-6, is highly expressed in aged mice as a consequence of an aberrant NF-*κ*B binding to miR-146a promoter. As a result, the negative feedback regulation loop inducing the downregulation of inflammatory factors can be interrupted in aged mice [103]. In macrophages isolated from aged mice, both DNA methyl-transferase inhibitor and histone deacetylase inhibitor are able to significantly upregulate miR-146a through transcriptional activation by altering DNA binding activity of NF-*κ*B [104]. Reduced miR-146a in oxLDL-activated macrophages is linked to an increase of its target, Toll-like receptor 4 (TLR4), involved in lipid uptake and inflammatory cytokine secretion [105].

Due to the upregulation in inflammatory condition in various primary disorders of skeletal muscle [106], miR-155 is identified as an important regulator of MEF2A expression and exhibits its function in myoblast differentiation [107], and it also plays a critical role in the regulation of inflammation that affects both innate and adaptive immunity. The upregulation of miR-155 is the most characteristic feature of the miRNA expression signature in LPS-stimulated macrophages through the binding to NF-*κ*B [108]. Moreover, the inflammatory response mediated by miR-155 is induced by TLR ligands and can enhance the translation of TNF-*α* during the pathogenesis of metabolic syndromes [109].

In addition, miR-23a negatively regulates PGC-1*α* as a key activator of mitochondrial biogenesis and function [110]. In another study, the level of miR-696 is upregulated in skeletal muscle of mice subjected to hind limb immobilization while the level of PGC-1*α* as the target of miR-696 exhibits an obvious decrease. Consistent with this observation, the overexpression of miR-696 in myocytes shows a decrease in PGC-1*α*, suggesting its involvement in mitochondrial function and metabolism, and its importance in controlling the metabolism, adaptation, and atrophy of skeletal muscle [111].

Since a large number of miRNAs have been identified in skeletal muscle, the investigation of miRNAs is a promising but relatively unexplored area for understanding the regulatory mechanisms of wasting process associated with cytokine expression and secretion in aging skeletal muscle. Clearly targeting these miRNAs could provide an efficient and noninvasive approach for the diagnosis, prevention, or treatment of sarcopenia through the regulation of proinflammatory and anti-inflammatory factors.

## 3. Diagnostic and Therapeutic Opportunity

Potential interventions for sarcopenia include nutritional supplements, physical activity/resistance exercise, caloric restriction, anabolic hormones, anti-inflammatory agents, and antioxidants. A key question is whether sarcopenia is a truly distinct syndrome or a milder form of a cachexia continuum [112]. It is difficult to estimate the prevalence of sarcopenia, mostly because of practical difficulties in assessing skeletal muscle mass [113]. Sarcopenia-associated difference in the production of proinflammatory cytokines* in vivo* shows a prolonged inflammation activity. There remains a lack of understanding of individual contributions of various cytokines during the aging process of skeletal muscle, because of their wide-ranging effects on cell proliferation, differentiation, migration, survival, and apoptosis. As reviewed here, miRNAs play major roles in the inflammatory regulation as the pivotal regulators for modulating cell functions or the critical factors for affecting the therapeutic outcome of sarcopenia. Although the central role of inflammation in sarcopenia and proinflammatory cytokines including TNF-*α* and IL-6 as the central mediators of skeletal muscle atrophy has been documented, the roles of inflammatory miRNAs in mass maintenance and functional development of skeletal muscle still need to be further explored and confirmed.

Whether miRNAs can be used for the diagnosis of inflammatory involvement in the aging process of skeletal muscle depends on the precise characterization, specific distribution, and accurate regulation of miRNAs. Proinflammatory cytokine-associated miRNAs such as miR-146a, miR-181, and miR-21 are frequently detected at a high level in skeletal muscle; however, their functions are still not fully clear. Therefore, further exploration of their targets, regulatory networks, and functions is highly desired. In conclusion, the discovery of miRNAs with regulatory capacity of inflammatory response during aging process of skeletal muscle will open a novel avenue for the diagnosis, prevention, and therapy of sarcopenia through effectively modulating inflammatory signal pathways.

## References

[1] Morley J. E., Anker S. D., von Haehling S. (2014). Prevalence, incidence, and clinical impact of sarcopenia: facts, numbers, and epidemiology—update 2014. *Journal of Cachexia, Sarcopenia and Muscle*.

[2] Fan J., Kou X., Jia S., Yang X., Yang Y., Chen N. (2016). Autophagy as a potential target for sarcopenia. *Journal of Cellular Physiology*.

[3] Kandarian S. C., Jackman R. W. (2006). Intracellular signaling during skeletal muscle atrophy. *Muscle and Nerve*.

[4] White J. R., Confides A. L., Moore-Reed S., Hoch J. M., Dupont-Versteegden E. E. (2015). Regrowth after skeletal muscle atrophy is impaired in aged rats, despite similar responses in signaling pathways. *Experimental Gerontology*.

[5] Kaasik P., Umnova M., Pehme A. (2007). Ageing and dexamethasone associated sarcopenia: peculiarities of regeneration. *Journal of Steroid Biochemistry and Molecular Biology*.

[6] Howard C., Ferrucci L., Sun K. (2007). Oxidative protein damage is associated with poor grip strength among older women living in the community. *Journal of Applied Physiology*.

[7] Miller R. A. (1996). The aging immune system: primer and prospectus. *Science*.

[8] Merritt E. K., Stec M. J., Thalacker-Mercer A. (2013). Heightened muscle inflammation susceptibility may impair regenerative capacity in aging humans. *Journal of Applied Physiology*.

[9] Lee J. S. W., Auyeung T.-W., Kwok T., Lau E. M. C., Leung P.-C., Woo J. (2008). Associated factors and health impact of sarcopenia in older Chinese men and women: a cross-sectional study. *Gerontology*.

[10] Cai D., Frantz J. D., Tawa N. E. (2004). IKK*β*/NF-*κ*B activation causes severe muscle wasting in mice. *Cell*.

[11] Adler A. S., Sinha S., Kawahara T. L. A., Zhang J. Y., Segal E., Chang H. Y. (2007). Motif module map reveals enforcement of aging by continual NF-*κ*B activity. *Genes & Development*.

[12] Budui S. L., Rossi A. P., Zamboni M. (2015). The pathogenetic bases of sarcopenia. *Clinical Cases in Mineral and Bone Metabolism*.

[13] Batsis J. A., Mackenzie T. A., Jones J. D., Lopez-Jimenez F., Bartels S. J. (2016). Sarcopenia, sarcopenic obesity and inflammation: results from the 1999–2004 National Health and Nutrition Examination Survey. *Clinical Nutrition*.

[14] Kim T. N., Choi K. M. (2015). The implications of sarcopenia and sarcopenic obesity on cardiometabolic disease. *Journal of Cellular Biochemistry*.

[15] Phillips C. M., Perry I. J. (2013). Does inflammation determine metabolic health status in obese and nonobese adults?. *Journal of Clinical Endocrinology and Metabolism*.

[16] Green D. R., Galluzzi L., Kroemer G. (2011). Mitochondria and the autophagy-inflammation-cell death axis in organismal aging. *Science*.

[17] Ko F., Abadir P., Marx R. (2016). Impaired mitochondrial degradation by autophagy in the skeletal muscle of the aged female interleukin 10 null mouse. *Experimental Gerontology*.

[18] Correia-Melo C., Marques F. D., Anderson R. (2016). Mitochondria are required for pro-ageing features of the senescent phenotype. *The EMBO Journal*.

[19] Kepp O., Galluzzi L., Kroemer G. (2011). Mitochondrial control of the NLRP3 inflammasome. *Nature Immunology*.

[20] Shimada K., Crother T. R., Karlin J. (2012). Oxidized mitochondrial DNA activates the NLRP3 inflammasome during apoptosis. *Immunity*.

[21] Li Q., Engelhardt J. F. (2006). Interleukin-1*β* induction of NF*κ*B is partially regulated by H_2_O_2_-mediated activation of NF*κ*B-inducing kinase. *The Journal of Biological Chemistry*.

[22] Fan C., Li Q., Zhang Y. (2004). IkappaBalpha and IkappaBbeta possess injury context-specific functions that uniquely influence hepatic NF-kappaB induction and inflammation. *The Journal of Clinical Investigation*.

[23] Sriram S., Subramanian S., Sathiakumar D. (2011). Modulation of reactive oxygen species in skeletal muscle by myostatin is mediated through NF-*κ*B. *Aging Cell*.

[24] Rhoads M. G., Kandarian S. C., Pacelli F., Doglietto G. B., Bossola M. (2010). Expression of NF-*κ*B and I*κ*B proteins in skeletal muscle of gastric cancer patients. *European Journal of Cancer*.

[25] White J. P., Puppa M. J., Gao S., Sato S., Welle S. L., Carson J. A. (2013). Muscle mTORC1 suppression by IL-6 during cancer cachexia: a role for AMPK. *American Journal of Physiology—Endocrinology and Metabolism*.

[26] McIntire K. L., Chen Y., Sood S., Rabkin R. (2014). Acute uremia suppresses leucine-induced signal transduction in skeletal muscle. *Kidney International*.

[27] He W. A., Berardi E., Cardillo V. M. (2013). NF-*κ*B-mediated Pax7 dysregulation in the muscle microenvironment promotes cancer cachexia. *The Journal of Clinical Investigation*.

[28] Wang B., Yang G., Liang X., Zhu M., Du M. (2014). Grape seed extract prevents skeletal muscle wasting in interleukin 10 knockout mice. *BMC Complementary and Alternative Medicine*.

[29] Peake J. M., Della Gatta P., Suzuki K., Nieman D. C. (2015). Cytokine expression and secretion by skeletal muscle cells: regulatory mechanisms and exercise effects. *Exercise Immunology Review*.

[30] Podbregar M., Lainscak M., Prelovsek O., Mars T. (2013). Cytokine response of cultured skeletal muscle cells stimulated with proinflammatory factors depends on differentiation stage. *The Scientific World Journal*.

[31] Molanouri Shamsi M., Hassan Z. H., Gharakhanlou R. (2014). Expression of interleukin-15 and inflammatory cytokines in skeletal muscles of STZ-induced diabetic rats: effect of resistance exercise training. *Endocrine*.

[32] Visser M., Pahor M., Taaffe D. R. (2002). Relationship of interleukin-6 and tumor necrosis factor-*α* with muscle mass and muscle strength in elderly men and women: the Health ABC Study. *Journals of Gerontology, Series A: Biological Sciences and Medical Sciences*.

[33] Giuliani N., Sansoni P., Girasole G. (2001). Serum interleukin-6, soluble interleukin-6 receptor and soluble gp130 exhibit different patterns of age- and menopause-related changes. *Experimental Gerontology*.

[34] Kristiansen O. P., Mandrup-Poulsen T. (2005). Interleukin-6 and diabetes: the good, the bad, or the indifferent?. *Diabetes*.

[35] Kamimura D., Ishihara K., Hirano T. (2003). IL-6 signal transduction and its physiological roles: the signal orchestration model. *Reviews of Physiology, Biochemistry and Pharmacology*.

[36] Ershler W. B., Keller E. T. (2000). Age-associated increased interleukin-6 gene expression, late-life diseases, and frailty. *Annual Review of Medicine*.

[37] Watkins S. K., Zhu Z., Riboldi E. (2011). FOXO3 programs tumor-associated DCs to become tolerogenic in human and murine prostate cancer. *The Journal of Clinical Investigation*.

[38] Tierney M. T., Aydogdu T., Sala D. (2014). STAT3 signaling controls satellite cell expansion and skeletal muscle repair. *Nature Medicine*.

[39] Green C. J., Macrae K., Fogarty S., Hardie D. G., Sakamoto K., Hundal H. S. (2011). Counter-modulation of fatty acid-induced pro-inflammatory nuclear factor *κ*B signalling in rat skeletal muscle cells by AMP-activated protein kinase. *Biochemical Journal*.

[40] Zhang L., Rajan V., Lin E. (2011). Pharmacological inhibition of myostatin suppresses systemic inflammation and muscle atrophy in mice with chronic kidney disease. *The FASEB Journal*.

[41] Carey A. L., Steinberg G. R., Macaulay S. L. (2006). Interleukin-6 increases insulin-stimulated glucose disposal in humans and glucose uptake and fatty acid oxidation in vitro via AMP-activated protein kinase. *Diabetes*.

[42] Bruce C. R., Dyck D. J. (2004). Cytokine regulation of skeletal muscle fatty acid metabolism: effect of interleukin-6 and tumor necrosis factor-*α*. *American Journal of Physiology—Endocrinology and Metabolism*.

[43] Franckhauser S., Elias I., Rotter Sopasakis V. (2008). Overexpression of Il6 leads to hyperinsulinaemia, liver inflammation and reduced body weight in mice. *Diabetologia*.

[44] Haddad F., Zaldivar F., Cooper D. M., Adams G. R. (2005). IL-6-induced skeletal muscle atrophy. *Journal of Applied Physiology*.

[45] Schaap L. A., Pluijm S. M. F., Deeg D. J. H., Visser M. (2006). Inflammatory markers and loss of muscle mass (sarcopenia) and strength. *The American Journal of Medicine*.

[46] Yang C.-W., Li C.-I., Li T.-C. (2015). Association of sarcopenic obesity with higher serum high-sensitivity c-reactive protein levels in Chinese older males—a community-based study (Taichung Community Health Study-Elderly, TCHS-E). *PLoS ONE*.

[47] Girven M., Dugdale H. F., Owens D. J., Hughes D. C., Stewart C. E., Sharples A. P. (2016). L-glutamine improves skeletal muscle cell differentiation and prevents myotube atrophy after cytokine (TNF-*α*) stress via reduced p38 MAPK signal transduction. *Journal of Cellular Physiology*.

[48] Otis J. S., Niccoli S., Hawdon N. (2014). Pro-inflammatory mediation of myoblast proliferation. *PLoS ONE*.

[49] Ciciliot S., Schiaffino S. (2010). Regeneration of mammalian skeletal muscle. Basic mechanisms and clinical implications. *Current Pharmaceutical Design*.

[50] Luo G., Hershko D. D., Robb B. W., Wray C. J., Hasselgren P.-O. (2003). IL-1*β* stimulates IL-6 production in cultured skeletal muscle cells through activation of MAP kinase signaling pathway and NF-*κ*B. *American Journal of Physiology—Regulatory Integrative and Comparative Physiology*.

[51] Wang D.-T., Yin Y., Yang Y.-J. (2014). Resveratrol prevents TNF-*α*-induced muscle atrophy via regulation of Akt/mTOR/FoxO1 signaling in C2C12 myotubes. *International Immunopharmacology*.

[52] Schaap L. A., Pluijm S. M. F., Deeg D. J. H. (2009). Higher inflammatory marker levels in older persons: associations with 5-year change in muscle mass and muscle strength. *Journals of Gerontology—Series A Biological Sciences and Medical Sciences*.

[53] Greiwe J. S., Bo C., Rubin D. C., Yarasheski K. E., Semenkovich C. F. (2001). Resistance exercise decreases skeletal muscle tumor necrosis factor *α* in frail elderly humans. *The FASEB Journal*.

[54] Mangner N., Linke A., Oberbach A. (2013). Exercise training prevents TNF-*α* induced loss of force in the diaphragm of mice. *PLoS ONE*.

[55] Zhao Q., Yang S. T., Wang J. J. (2015). TNF alpha inhibits myogenic differentiation of C2C12 cells through NF-*κ*B activation and impairment of IGF-1 signaling pathway. *Biochemical and Biophysical Research Communications*.

[56] Coletti D., Moresi V., Adamo S., Molinaro M., Sassoon D. (2005). Tumor necrosis factor-*α* gene transfer induces cachexia and inhibits muscle regeneration. *Genesis*.

[57] Frost R. A., Lang C. H. (2007). Protein kinase B/Akt: a nexus of growth factor and cytokine signaling in determining muscle mass. *Journal of Applied Physiology*.

[58] Lang C. H., Frost R. A., Vary T. C. (2007). Regulation of muscle protein synthesis during sepsis and inflammation. *American Journal of Physiology—Endocrinology and Metabolism*.

[59] Di Renzo L., Sarlo F., Petramala L. (2013). Association between -308 G/A TNF- *α* polymorphism and appendicular skeletal muscle mass index as a marker of sarcopenia in normal weight obese syndrome. *Disease Markers*.

[60] Pijet B., Pijet M., Litwiniuk A., Gajewska M., Pająk B., Orzechowski A. (2013). TNF-*α* and IFN-s-dependent muscle decay is linked to NF-*κ*B- and STAT-1*α*-stimulated Atrogin1 and MuRF1 genes in C2C12 myotubes. *Mediators of Inflammation*.

[61] Quinn L. S., Anderson B. G., Strait-Bodey L., Stroud A. M., Argués J. M. (2009). Oversecretion of interleukin-15 from skeletal muscle reduces adiposity. *American Journal of Physiology—Endocrinology and Metabolism*.

[62] Boström P., Wu J., Jedrychowski M. P. (2012). A PGC1-*α*-dependent myokine that drives brown-fat-like development of white fat and thermogenesis. *Nature*.

[63] Seldin M. M., Wong G. W. (2014). Regulation of tissue crosstalk by skeletal muscle-derived myonectin and other myokines. *Adipocyte*.

[64] Steensberg A., Fischer C. P., Keller C., Møller K., Pedersen B. K. (2003). IL-6 enhances plasma IL-1ra, IL-10, and cortisol in humans. *American Journal of Physiology—Endocrinology and Metabolism*.

[65] Drummond M. J., Timmerman K. L., Markofski M. M. (2013). Short-term bed rest increases TLR4 and IL-6 expression in skeletal muscle of older adults. *American Journal of Physiology—Regulatory Integrative and Comparative Physiology*.

[66] Eisele P. S., Furrer R., Beer M., Handschin C. (2015). The PGC-1 coactivators promote an anti-inflammatory environment in skeletal muscle in vivo. *Biochemical and Biophysical Research Communications*.

[67] Sczelecki S., Besse-Patin A., Abboud A. (2014). Loss of Pgc-1*α* expression in aging mouse muscle potentiates glucose intolerance and systemic inflammation. *American Journal of Physiology—Endocrinology and Metabolism*.

[68] Eisele P. S., Salatino S., Sobek J., Hottiger M. O., Handschin C. (2013). The peroxisome proliferator-activated receptor *γ* coactivator 1*α*/*β* (PGC-1) coactivators repress the transcriptional activity of NF-*κ*B in skeletal muscle cells. *Journal of Biological Chemistry*.

[69] Wenz T., Rossi S. G., Rotundo R. L., Spiegelman B. M., Moraes C. T. (2009). Increased muscle PGC-1*α* expression protects from sarcopenia and metabolic disease during aging. *Proceedings of the National Academy of Sciences of the United States of America*.

[70] Bushati N., Cohen S. M. (2007). MicroRNA functions. *Annual Review of Cell and Developmental Biology*.

[71] Zacharewicz E., Lamon S., Russell A. P. (2013). MicroRNAs in skeletal muscle and their regulation with exercise, ageing, and disease. *Frontiers in Physiology*.

[72] Guo H., Ingolia N. T., Weissman J. S., Bartel D. P. (2010). Mammalian microRNAs predominantly act to decrease target mRNA levels. *Nature*.

[73] Han J., Lee Y., Yeom K.-H. (2006). Molecular basis for the recognition of primary microRNAs by the Drosha-DGCR8 complex. *Cell*.

[74] Diebel K. W., Claypool D. J., van Dyk L. F. (2014). A conserved RNA polymerase III promoter required for gammaherpesvirus TMER transcription and microRNA processing. *Gene*.

[75] Winter J., Jung S., Keller S., Gregory R. I., Diederichs S. (2009). Many roads to maturity: MicroRNA biogenesis pathways and their regulation. *Nature Cell Biology*.

[76] Cui Y., Huang T., Zhang X. (2015). RNA editing of microRNA prevents RNA-induced silencing complex recognition of target mRNA. *Open Biology*.

[77] Brodersen P., Voinnet O. (2009). Revisiting the principles of microRNA target recognition and mode of action. *Nature Reviews Molecular Cell Biology*.

[78] Hu Z., Bruno A. E. (2011). The influence of 3′UTRs on MicroRNA function inferred from human SNP data. *Comparative and Functional Genomics*.

[79] Kim Y., Kim B., Kim V. N. (2016). Re-evaluation of the roles of DROSHA, Exportin 5, and DICER in microRNA biogenesis. *Proceedings of the National Academy of Sciences*.

[80] Brennecke J., Stark A., Russell R. B., Cohen S. M. (2005). Principles of microRNA-target recognition. *PLoS Biology*.

[81] Quattrocelli M., Sampaolesi M. (2015). The mesmiRizing complexity of microRNAs for striated muscle tissue engineering. *Advanced Drug Delivery Reviews*.

[82] Baltimore D., Boldin M. P., O'Connell R. M., Rao D. S., Taganov K. D. (2008). MicroRNAs: new regulators of immune cell development and function. *Nature Immunology*.

[83] van Rooij E., Liu N., Olson E. N. (2008). MicroRNAs flex their muscles. *Trends in Genetics*.

[84] Small E. M., O'Rourke J. R., Moresi V. (2010). Regulation of PI3-kinase/Akt signaling by muscle-enriched microRNA-486. *Proceedings of the National Academy of Sciences of the United States of America*.

[85] Callis T. E., Deng Z., Chen J.-F., Wang D.-Z. (2008). Muscling through the microRNA world. *Experimental Biology and Medicine*.

[86] van Rooij E., Quiat D., Johnson B. A. (2009). A family of microRNAs encoded by myosin genes governs myosin expression and muscle performance. *Developmental Cell*.

[87] Chen J.-F., Callis T. E., Wang D.-Z. (2009). microRNAs and muscle disorders. *Journal of Cell Science*.

[88] Nakasa T., Ishikawa M., Shi M., Shibuya H., Adachi N., Ochi M. (2010). Acceleration of muscle regeneration by local injection of muscle-specific microRNAs in rat skeletal muscle injury model. *Journal of Cellular and Molecular Medicine*.

[89] Drummond M. J., McCarthy J. J., Fry C. S., Esser K. A., Rasmussen B. B. (2008). Aging differentially affects human skeletal muscle microRNA expression at rest and after an anabolic stimulus of resistance exercise and essential amino acids. *American Journal of Physiology—Endocrinology and Metabolism*.

[90] Contreras J., Rao D. S. (2012). MicroRNAs in inflammation and immune responses. *Leukemia*.

[91] Schroen B., Heymans S. (2012). Small but smartmicroRNAs in the centre of inflammatory processes during cardiovascular diseases, the metabolic syndrome, and ageing. *Cardiovascular Research*.

[92] Mercken E. M., Majounie E., Ding J. (2013). Age-associated miRNA alterations in skeletal muscle from rhesus monkeys reversed by caloric restriction. *Aging*.

[93] Li Q.-J., Chau J., Ebert P. J. R. (2007). miR-181a is an intrinsic modulator of T cell sensitivity and selection. *Cell*.

[94] Soriano-Arroquia A., House L., Tregilgas L., Canty-Laird E., Goljanek-Whysall K. (2016). The functional consequences of age-related changes in microRNA expression in skeletal muscle. *Biogerontology*.

[95] Xie W., Li Z., Li M., Xu N., Zhang Y. (2013). miR-181a and inflammation: miRNA homeostasis response to inflammatory stimuli in vivo. *Biochemical and Biophysical Research Communications*.

[96] Georgantas R. W., Streicher K., Greenberg S. A. (2014). Inhibition of myogenic microRNAs 1, 133, and 206 by inflammatory cytokines links inflammation and muscle degeneration in adult inflammatory myopathies. *Arthritis and Rheumatology*.

[97] Yadava R. S., Foff E. P., Yu Q. (2015). TWEAK/Fn14, a pathway and novel therapeutic target in myotonic dystrophy. *Human Molecular Genetics*.

[98] Panguluri S. K., Bhatnagar S., Kumar A. (2010). Genomic profiling of messenger RNAs and microRNAs reveals potential mechanisms of TWEAK-induced skeletal muscle wasting in mice. *PLoS ONE*.

[99] Roush S., Slack F. J. (2008). The let-7 family of microRNAs. *Trends in Cell Biology*.

[100] Drummond M. J., McCarthy J. J., Sinha M. (2011). Aging and microRNA expression in human skeletal muscle: a microarray and bioinformatics analysis. *Physiological Genomics*.

[101] Jiang L. Q., Franck N., Egan B. (2013). Autocrine role of interleukin-13 on skeletal muscle glucose metabolism in type 2 diabetic patients involves microRNA let-7. *American Journal of Physiology—Endocrinology and Metabolism*.

[102] Yu J. H., Long L., Luo Z. X., Li L. M., You J. R. (2016). Anti-inflammatory role of microRNA let-7c in LPS treated alveolar macrophages by targeting STAT3. *Asian Pacific Journal of Tropical Medicine*.

[103] Jiang M., Xiang Y., Wang D. (2012). Dysregulated expression of miR-146a contributes to age-related dysfunction of macrophages. *Aging Cell*.

[104] Olivieri F., Rippo M. R., Prattichizzo F. (2013). Toll like receptor signaling in ‘inflammaging’: microRNA as new players. *Immunity and Ageing*.

[105] Schulte L. N., Westermann A. J., Vogel J. (2013). Differential activation and functional specialization of miR-146 and miR-155 in innate immune sensing. *Nucleic Acids Research*.

[106] Eisenberg I., Eran A., Nishino I. (2007). Distinctive patterns of microRNA expression in primary muscular disorders. *Proceedings of the National Academy of Sciences of the United States of America*.

[107] Seok H. Y., Tatsuguchi M., Callis T. E., He A., Pu W. T., Wang D.-Z. (2011). miR-155 inhibits expression of the MEF2A protein to repress skeletal muscle differentiation. *The Journal of Biological Chemistry*.

[108] Taganov K. D., Boldin M. P., Chang K.-J., Baltimore D. (2006). NF-*κ*B-dependent induction of microRNA miR-146, an inhibitor targeted to signaling proteins of innate immune responses. *Proceedings of the National Academy of Sciences of the United States of America*.

[109] Tili E., Michaille J.-J., Cimino A. (2007). Modulation of miR-155 and miR-125b levels following lipopolysaccharide/TNF- *α* stimulation and their possible roles in regulating the response to endotoxin shock. *Journal of Immunology*.

[110] Russell A. P., Wada S., Vergani L. (2013). Disruption of skeletal muscle mitochondrial network genes and miRNAs in amyotrophic lateral sclerosis. *Neurobiology of Disease*.

[111] Aoi W., Naito Y., Mizushima K. (2010). The microRNA miR-696 regulates PGC-1*α* in mouse skeletal muscle in response to physical activity. *American Journal of Physiology—Endocrinology and Metabolism*.

[112] Jensen G. L. (2008). Inflammation: roles in aging and sarcopenia. *Journal of Parenteral and Enteral Nutrition*.

[113] Villani A. M., Crotty M., Cameron I. D. (2014). Appendicular skeletal muscle in hospitalised hip-fracture patients: development and cross-validation of anthropometric prediction equations against dual-energy X-ray absorptiometry. *Age and Ageing*.

